# Search Volume of Insomnia and Suicide as Digital Footprints of Global Mental Health During the COVID-19 Pandemic: 3-Year Infodemiology Study

**DOI:** 10.2196/67646

**Published:** 2025-04-17

**Authors:** Sheng-Hsuan Lin, Kuan-Pin Su, Hsiao-Hui Tsou, Pei-Hsuan Hsia, Yu-Hsuan Lin

**Affiliations:** 1 Institute of Statistics National Yang Ming Chiao Tung University Hsinchu Taiwan; 2 Institute of Data Science and Engineering National Yang Ming Chiao Tung University Hsinchu Taiwan; 3 Institute of Applied Mathematics National Dong Hwa University Hualien Taiwan; 4 Institute of Biochemical and Molecular Medical Sciences National Dong Hwa University Hualien Taiwan; 5 An-Nan Hospital China Medical University Tainan Taiwan; 6 College of Medicine China Medical University Taichung Taiwan; 7 Mind-Body Interface Research Center (MBI-Lab) China Medical University Hospital Taichung Taiwan; 8 Institute of Population Health Sciences National Health Research Institutes Miaoli County Taiwan; 9 Department of Psychiatry College of Medicine National Taiwan University Taipe Taiwan; 10 Department of Biomedical Sciences and Engineering National Central University Taoyuan Taiwan; 11 Department of Psychiatry National Taiwan University Hospital Taipei Taiwan

**Keywords:** mediation analysis, internet searches, stay-at-home measures, insomnia, suicide, COVID-19

## Abstract

**Background:**

The global COVID-19 pandemic’s mental health impact was primarily studied in the initial year of lockdowns but remained underexplored in subsequent years despite evolving conditions. This study aimed to address this gap by investigating how COVID-19–related factors, including nationwide COVID-19 deaths and incidence rates, influenced mental health indicators over time.

**Objective:**

This study aimed to examine the interplay among national COVID-19 pandemic deaths, incidence rates, stay-at-home behaviors, and mental health indicators across different income-level countries. Specifically, we assessed the mediating role of stay-at-home behaviors in the relationship between the COVID-19 pandemic deaths and mental health indicators.

**Methods:**

We analyzed data from 45 countries spanning March 2020 to October 2022. COVID-19–related factors included national COVID-19 pandemic deaths and incidence rates, obtained from publicly available datasets. Stay-at-home behaviors were assessed using Google Location History data, which captured residence-based cell phone activity as a proxy for mobility patterns. Mental health indicators were evaluated through Google Trends data, measuring changes in search volumes for “insomnia” and “suicide.” The interplay among these variables was assessed using mediation analysis to quantify the proportion mediated by stay-at-home behaviors in the association between COVID-19 deaths and mental health indicators.

**Results:**

In high-income countries, during the first pandemic year (March 2020 to February 2021), a higher monthly COVID-19 death count was associated with increased searches for “insomnia,” with a total effect estimate of 2.1×10^-4^ (95% CI 4.3×10^-5^ to 3.9×10^-4^; *P*=.01). Stay-at-home behaviors mediated 31.9% of this effect (95% CI 9.8% to 127.5%, *P*=.02). This association weakened and became nonsignificant in the second and third years (*P*=.25 and *P*=.54, respectively). For middle-income countries, a different pattern emerged regarding “suicide” searches. Higher COVID-19 death counts were linked to a decline in “suicide” searches in the first (estimate: –3.5×10^-4^, 95% CI –6.1×10^-4^ to –9.8×10^-5^; *P*=.006) and second years (*P*=.01). Mediation analysis indicated that this effect was not significantly explained by stay-at-home behaviors, suggesting the influence of other societal factors. In high-income countries, no significant association between COVID-19 deaths and “suicide” searches was observed in the first year (*P*=.86). However, a positive association emerged in the second year, approaching statistical significance (estimate: 2.2×10^-4^, 95% CI –9.5×10^-7^ to 4.2×10^-4^; *P*=.05), and became significant in the third year (estimate: 5.0×10^-4^, 95% CI 5.0×10^-5^ to 1.0×10^-3^; *P*=.03,), independent of stay-at-home behaviors.

**Conclusions:**

Our findings highlight how the mental health impact of the pandemic varied across income groups and evolved over time. The mediating effect of stay-at-home behaviors was significant in the early phases but diminished in later stages, particularly in high-income countries. Meanwhile, middle-income countries exhibited unique patterns that suggest alternative protective factors. These insights can inform tailored mental health interventions and policy strategies in future public health crises.

## Introduction

The COVID-19 pandemic profoundly reshaped global mental health, with emerging evidence highlighting its widespread impact. Studies reported a marked increase in sleep disturbances, depression, and anxiety during this period [1-4]. A meta-analysis showed a 27.6% rise in global major depressive disorder cases and a 25.6% increase in anxiety disorders, closely associated with pandemic-related factors such as infection rates and reduced mobility [5]. However, an apparent paradox was observed in suicide rates: despite the heightened prevalence of depression and anxiety, a large-scale multinational survey spanning the pandemic’s initial 9-15 months (up to June 2021) revealed that global suicide rates remained stable [6]. This stability persisted even when factors such as the COVID-19 pandemic mortality rates, public health response stringency, and economic support levels were examined. The findings underscore the complex and multifactorial relationship between the pandemic and mental health outcomes. Furthermore, insomnia (a condition intricately linked to stress, depression, and anxiety) emerged as a potential early marker for these mental health conditions [7], emphasizing the need for targeted monitoring and intervention strategies.

As the pandemic unfolded, digital data sources became invaluable for capturing population-level behavioral changes in real time. Among these tools, Google Trends emerged as a robust method for monitoring public and mental health trends across diverse contexts. Its high temporal resolution and global reach allowed researchers to track nuanced behavioral patterns, such as the onset of influenza outbreaks and long-term depression risk factors [8,9]. During the pandemic’s early stages, a marked increase in internet searches for “insomnia” was consistently observed, reflecting shifts in mental health dynamics across several countries [10-13]. Building on this foundation, our study explored the relationship between national COVID-19 death rates and search patterns for “insomnia” and “suicide” during the pandemic’s first year [11]. In addition, we investigated the role of stay-at-home measures in 45 countries by analyzing correlations between search trends and residential cell phone activity. Our findings indicated a significant mediation effect of stay-at-home behaviors on COVID-19–related insomnia during the first 6 months of the pandemic, though the results for suicide-related searches were more varied. These insights underscore the importance of refining methodologies to better understand the broader implications of pandemic-related restrictions on mental health indicators [11].

While early pandemic research provided valuable insights into the mental health impacts of strict lockdowns and stay-at-home measures, most studies were limited to the pandemic’s initial year [14]. However, this narrow focus risks overlooking the evolving dynamics of the pandemic’s longer-term effects on mental health. For instance, while early studies reported increased psychiatric risks among patients with COVID-19 [4], subsequent analyses of 2-year cohort studies revealed that the surge in mood and anxiety disorders was transient and comparable to other respiratory infections [15]. This discrepancy highlights the need for a more extended temporal perspective to capture the interplay of factors such as vaccine distribution, new viral variants, and shifting public health measures. By extending our research to encompass 3 years of the pandemic, we aimed to provide a more comprehensive understanding of its mental health impact. Our study focused on key factors, including COVID-19 deaths, incidence rates, stay-at-home behaviors, and national income levels, to investigate their differential effects on mental health indicators. This approach allowed us to address limitations in previous research and contribute to a deeper understanding of the complex relationships between pandemic-related stressors and psychological outcomes.

## Methods

### Study Design and Data Collection

**Figure 1:**
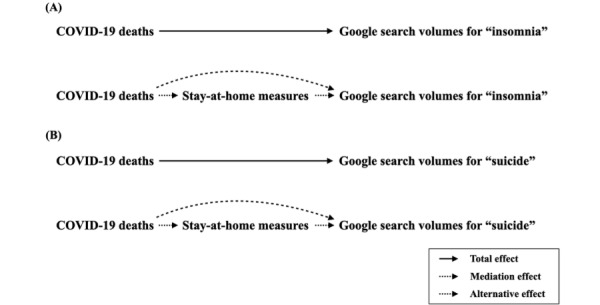
(A) Causal pathway illustrating COVID-19 deaths, stay-at-home behaviors, and search volumes for “insomnia,” and (B) “suicide”.

This study investigated the intricate relationship among monthly national COVID-19 deaths, stay-at-home behaviors, and internet searches for “insomnia” and “suicide” in local languages across 45 countries throughout the initial 3-year period of the COVID-19 pandemic (March 1, 2020, to October 15, 2022). Google Trends data were used to analyze changes in internet search volumes for the terms “insomnia” and “suicide” as indicators of mental health impacts. In addition, Google Location History data were used to estimate the impact of stay-at-home behaviors based on the timing of cell phone activity at residences (Figure 1). The study also quantified the proportion mediated (PM) by stay-at-home behaviors of COVID-19’s effects on insomnia and suicide ideation.

### Assessment of Mental Health Impacts at the Population Level

To gauge the mental health status of the general population across the 45 countries, search volumes for “insomnia” and “suicide” were retrieved from Google Trends data, spanning from March 1, 2015, to October 15, 2022. While search terms related to depression and anxiety have indeed been used as indicators of mental health in some studies [9,12], we chose to focus on insomnia and suicide for cross-national comparisons due to their broader recognition and more consistent meaning across different languages and cultures.

In contrast, the terms depression and anxiety may carry varying connotations across linguistic and cultural contexts, making direct comparisons more challenging. For example, in some cultures, depression may be understood primarily in a clinical sense, whereas in others, it might encompass a wider range of emotional distress. Similarly, anxiety might be associated with general stress in some languages but recognized as a specific psychological condition in others. These variations introduce potential inconsistencies when analyzing global search trends. Given that insomnia and suicide are more universally understood with relatively stable meanings, they are commonly used in cross-country research as reliable indicators of mental health trends [11-13]. Our approach aligns with previous studies that have utilized these terms for international comparisons.

Ensuring accurate translations of these terms into the local languages of the 45 countries, translations from both Chinese and English were cross-verified and back-translated using Google Translate [12,16]. Translated keywords for “insomnia” and “suicide,” along with the stay-at-home behaviors from March 1, 2020, to October 15, 2022, are summarized in Table 1. Weekly search volumes were analyzed between March 1, 2020, and October 15, 2022, and compared against expected search volumes derived from a 5-year baseline period: March 1, 2015, to February 28, 2020. This selection aimed to account for seasonal fluctuations in search volume.

Using search volumes from March 1, 2015, to February 28, 2020, for both keywords (“insomnia” and “suicide”), a counterfactual scenario was constructed to estimate expected search volumes in the absence of the COVID-19 outbreak. Expected search volumes were calculated through Hyndman and Khandakar’s algorithm for seasonal autoregressive integrated moving average (sARIMA) modeling [17]. The alteration in “insomnia” search volume was assessed by the difference between observed and expected volumes on a weekly basis from March 1, 2020, to October 15, 2022. A similar approach was applied to analyze “suicide” (Figure 1). In the following sections, the term “mental health impacts” describes observed changes in search volumes.

**Table 1:** Translations of the keywords “insomnia” and “suicide” for the 45 countries under study, and stay-at-home behaviors differentiated by the first year, second year, and third year from March 1, 2020, to October 2022. Stay-at-home behaviors were quantified as the increase relative to the baseline period of January 3 to February 6, 2020, as predefined by the Google Location History database. The numerical values (%) in the table represent the mean (95% CI).

Country		Translated keyword for insomnia	Translated keyword for suicide	The first year (March 2020 to February 2021)	The second year (March 2021 to February 2022)	The third year (March 2022 to October 2022)
**High income**						
	Australia	Insomnia	Suicide	8.52 (0.39 to 16.64)^a^	7.51 (0.56 to 14.47)^a^	4.88 (3.57 to 6.20)^a^
	Austria	Schlaflosigkeit	Selbstmord	8.12 (–3.82 to 20.06)	4.88 (–2.39 to 12.15)	4.88 (–2.39 to 12.15)
	Bolivia	Insomnio	Suicidio	19.16 (–1.26 to 39.58)	6.95 (2.48 to 11.41)^a^	7.39 (4.27 to 10.50)^a^
	Canada	Insomnia	Suicide	12.17 (3.20 to 21.14)^a^	8.85 (2.78 to 14.92)^a^	4.33 (1.02 to 7.65)^a^
	Chile	Insomnio	Suicidio	18.84 (7.35 to 30.34)^a^	12.89 (3.09 to 22.69)^a^	8.31 (4.51 to 12.11)^a^
	Czech republic	Nespavost	Sebevražda	7.12 (–4.07 to 18.32)	4.36 (–4.64, 13.35)	0.82 (–2.67, 4.32)
	Finland	Unettomuus	Itsemurha	6.09 (–2.64 to 14.81)	5.42 (–1.49 to 12.32)	2.87 (–1.21 to 6.95)
	France	Insomnie	Suicide	10.68 (–3.95 to 25.32)	4.97 (–1.36 to 11.31)	2.52 (–0.19 to 5.24)
	Germany	Schlaflosigkeit	Selbstmord	7.83 (–0.64 to 16.30)	5.99 (0.33 to 11.65)^a^	2.94 (0.17 to 5.71)^a^
	Greece	αυπνία	αυτοκτονία	7.63 (–7.36 to 22.63)	0.87 (–9.17 to 10.92)	–0.94 (–5.38 to 3.49)
	Hong Kong	失眠	自殺	11.64 (5.61 to 17.67)^a^	7.25 (–0.03 to 14.53)	9.79 (0.13 to 19.45)^a^
	Hungary	inszomnia	öngyilkosság	7.13 (–2.66 to 16.93)	4.06 (–3.59 to 11.71)	0.98 (1.98 to 3.93)
	Israel	**נדודי** **שינה**	**התאבדות**	12.52 (1.49 to 23.56)^a^	4.68 (–0.21 to 9.58)	3.38 (0.37 to 6.40)^a^
	Italy	Insonnia	Suicidio	10.83 (–6.45 to 28.12)	4.42 (–4.03 to 12.87)	1.63 (–1.73 to 4.99)
	Japan	不眠症	自殺	7.47 (1.32 to 13.63)^a^	6.68 (3.08 to 10.28)^a^	5.47 (2.59 to 8.36)^a^
	The Netherlands	Slapeloosheid	Zelfmoord	9.48 (2.24 to 16.72)^a^	6.51 (1.10 to 11.92)^a^	2.33 (0.08 to 4.59)^a^
	New Zealand	Insomnia	Mate whakamomori	7.75 (–7.77 to 23.28)	7.87 (–1.27 to 17.00)	6.79 (3.78, 9.80)^a^
	Norway	Søvnløs	Selvmord	6.44 (–1.86 to 14.96)	4.96 (–1.16 to 11.08)	1.17 (–1.42 to 3.75)
	Poland	Bezsenność	Samobójstwo	6.64 (4.10 to 17.37)	2.59 (–4.62 to 9.79)	0.84 (–1.83 to 3.50)
	Portugal	Insônia	Suicídio	14.32 (0.09 to 28.55)^a^	6.77 (–1.01 to 14.55)	4.13 (2.03 to 6.23)^a^
	Qatar	**الأرق**	**انتحار**	9.73 (–6.39 to 25.84)	1.43 (–4.47 to 7.32)	5.25 (1 to 9.49)^a^
	Saudi Arabia	**الأرق**	**انتحار**	11.67 (–2.46 to 25.81)	5.34 (2.56 to 8.13)^a^	7.90 (4.44 to 11.37)^a^
	Singapore	Insomnia	Suicide	19.73 (0.88 to 38.57)^a^	16.10 (9.73 to 22.47)^a^	9.99 (6.92 to 13.05)^a^
	South Korea	불면증	자살	4.92 (–1.03 to 10.88)	3.33 (–0.77 to 7.43)	1.30 (–3.73 to 6.34)
	Spain	Insomnio	Suicidio	10.75 (–4.11 to 25.60)	2.91 (–1.88 to 7.69)	0.48 (–2.12 to 3.08)
	Sweden	Sömnlös	Självmord	6.84 (0.36 to 13.33)^a^	5.66 (0.45 to 10.88)^a^	2.14 (–0.17 to 4.46)
	Switzerland	Schlafstörung	Selbstmord	8.86 (–1.70 to 19.42)	5.54 (–0.38 to 11.47)	1.64 (–0.97 to 4.25)
	Taiwan	失眠	自殺	1.76 (–0.66 to 4.18)	6.36 (–4.16 to 16.89)	6.02 (1.28 to 10.75)^a^
	United Arab Emirates	**الأرق**	**انتحار**	13.05 (–1.41 to 27.51)	6.47 (2.38 to 10.56)^a^	7.83 (4.20 to 11.46)^a^
	United Kingdom	Insomnia	Suicide	14.30 (3.38 to 25.23)^a^	7.94 (2.63 to 13.25)^a^	4.16 (2.71 to 5.61)^a^
	United States	Insomnia	Suicide	10.09 (3.48 to 16.71)^a^	5.63 (2.56 to 8.70)^a^	3.57 (2.08 to 5.06)^a^
**Middle income**						
	Argentina	Insomnio	Suicidio	13.99 (1.91 to 26.08)^a^	5.10 (–2.14 to 12.34)	2.91 (1.33 to 4.49)^a^
	Brazil	Insônia	Suicídio	9.95 (2.00 to 17.89)^a^	5.98 (0.66 to 11.30)^a^	3.35 (1.80 to 4.90)^a^
	Colombia	Insomnio	Suicidio	16.48 (2.11 to 30.85)^a^	6.73 (–0.86 to 14.33)	3.33 (1.47 to 5.20)^a^
	Egypt	**الأرق**	**انتحار**	6.43 (–3.26, to 16.11)	–2.33 (–7.14 to 2.47)	4.26 (0.71 to 7.81)^a^
	India	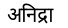	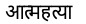	13.93 (1.78 to 26.07)^a^	11.47 (1.53 to 21.41)^a^	11.98 (9.89 to 14.07)^a^
	Indonesia	Insomnia	Bunuh diri	10.90 (4.04 to 17.75)^a^	6.96 (2.11 to 11.80)^a^	14.56 (12.51 to 16.62)^a^
	Malaysia	Insomnia	Membunuh diri	15.44 (–1.32 to 32.20)	17.12 (0.86 to 33.37)^a^	10.65 (7.13 to 14.16)^a^
	Mexico	Insomnio	Suicidio	12.70 (4.27 to 21.12)^a^	5.98 (2.80 to 9.16)^a^	3.77 (1.42 to 6.12)^a^
	Peru	Insomnio	Suicidio	22.56 (7.36 to 37.77)^a^	13.53 (7.94 to 19.11)^a^	9.42 (6.81 to 12.03)^a^
	South Africa	Insomnia	Suicide	15.80 (1.18 to 30.41)^a^	13.39 (7.56 to 19.22)^a^	13.75 (10.88 to 16.63)^a^
	Thailand	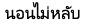	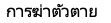	5.82 (–4.89 to 16.54)	4.20 (–3.80 to 12.19)	8.72 (6.10 to 11.35)^a^
	Turkey	Uykusuzluk	Intihar	8.78 (–6.46 to 24.02)	3.12 (–7.12 to 13.37)	0.37 (–3.21 to 3.95)
	Venezuela	Insomnio	Suicidio	14.13 (7.47 to 20.78)^a^	8.21 (3.69 to 12.74)^a^	12.00 (9.43 to 14.58)^a^
	Vietnam	mất ngủ	tự tử	1.47 (–12.80 to 15.73)	1.94 (–17.49 to 21.36)	6.76 (4.53 to 9)^a^

^a^These values indicate statistically significant differences compared with the baseline values.

### Quantification of Stay-at-Home Behaviors

This study used aggregated and anonymized cell phone location data provided by Google from users who opted in to share their location data through Google Location History. These data represent changes in mobility patterns across various locations, including residential, workplaces, retail and recreation, grocery stores and pharmacies, parks, and transit stations. For the purposes of this study, we concentrated on cell phone activity at residences to measure the degree of stay-at-home behaviors, referred to as “stay-at-home measures.” The percentage change in time spent at residences compared with a baseline period was calculated to quantify these behaviors [18].

The baseline activity was defined as the average of median values during a 5-week period from January 3 to February 6, 2020, as predetermined by Google Location History. This baseline serves as a reference point for calculating percentage changes in mobility across various categories. The data is aggregated at the country or regional level, ensuring user anonymity, and is updated daily, enabling the analysis of mobility patterns during specific phases of the COVID-19 pandemic, such as lockdowns or easing of restrictions [18].

The methodology involves using cell phone location signals to determine the duration of time users spend at residential locations. The aggregated data is presented as percentage increases or decreases relative to the baseline, allowing researchers to track trends in stay-at-home behaviors across different regions. Importantly, the reports account for variations in smartphone penetration and regional behaviors, making them applicable in both high-income and low- to middle-income countries [19]. We analyzed the percentage of time spent at residences for 45 countries between March 1, 2020, and October 15, 2022. While data were available for complete 12-month periods for the first 2 years of the pandemic (March 1, 2020, to February 28, 2021, and March 1, 2021, to February 28, 2022), only data from March 1, 2022, to October 15, 2022, were available for the third year due to the discontinuation of open access data by Google Location History [18].

The data provided an opportunity to explore stay-at-home behaviors even in countries with lower smartphone penetration rates. For example, countries where approximately one-third of adults own smartphones have used similar methods to measure stay-at-home behaviors as part of COVID-19–related mobility analyses [19]. Previous studies in low- and middle-income countries demonstrated the utility of Google mobility data for assessing mobility changes during government-mandated restrictions [19]. These studies highlighted the relevance of aggregated mobility data for guiding public health responses and prioritizing interventions [19-21]. In Nigeria, for instance, mobility data were linked with closures and restrictions, showing a significant decrease in mobility across retail, workplaces, and transit stations while observing increased residential mobility during lockdowns [20]. Similarly, a study in Eswatini used mobility data to identify disease-driving factors and produce risk maps for COVID-19 [21]. These examples support the robustness of Google mobility data in capturing stay-at-home behaviors in diverse socioeconomic contexts.

### Assessment of the COVID-19 Spread Rate

To gauge the speed at which COVID-19 spread within each country during the study period, daily death counts were recorded and averaged on a monthly basis [22]. Death counts were chosen over confirmed case numbers due to limited population testing availability in most countries, especially those facing rapid COVID-19 outbreaks. To maintain uniformity, the absolute number of deaths, rather than deaths per population, was used as an indicator of COVID-19’s impact across all countries [11,12].

To further assess the robustness of our findings, we also conducted a sensitivity analysis using the COVID-19 incidence rate as an alternative measure of the pandemic’s impact (Multimedia Appendix 1). While COVID-19 deaths provide a direct indicator of disease severity, the incidence rate captures broader transmission dynamics, accounting for variations in population testing capacity and case ascertainment across countries. This approach aligns with previous large-scale systematic reviews and meta-analyses examining the mental health effects of the pandemic [5], where national infection rates have been commonly used as a measure of outbreak severity. By incorporating both indicators, we aimed to enhance the precision of our analysis and ensure the reliability of our conclusions.

### Statistical Analysis

Causal mediation analysis was conducted to explore the role of stay-at-home measures in the mechanism linking COVID-19 spread and mental health impacts, namely insomnia and suicide [23]. The exposure and outcome variables were COVID-19 spread and mental health impacts, respectively. Stay-at-home measures served as the mediator of interest between the causal effect of COVID-19 spread and mental health. A mediation model between these 3 variables was assumed (Figure 1). Causal mediation analysis decomposed the total effect of COVID-19 spread on mental health impacts into two parts: (1) the part involving changes in stay-at-home measures (mediation effect) and (2) the part unrelated to changes in stay-at-home measures (alternative effect, which is also termed as “direct effect” in literatures) [23]. The proportion mediated, indicating the extent of mediation involving stay-at-home measures, was calculated as the ratio of the mediation effect to the total effect. To assess potential effect modifications during different time periods, data were stratified, and results were analyzed for the first year (March 1, 2020, to February 28, 2021), second year (March 1, 2021, to February 28, 2022), and third year (March 1, 2022, to October 15, 2022) of the study's COVID-19 period. In addition, the data were also stratified by the countries’ income level [24].

Causal mediation analysis involved constructing 3 panel regression models to examine the relationship between COVID-19 deaths, stay-at-home measures, and mental health–related search behaviors. The first model assessed the overall impact of COVID-19 deaths on the search volume for insomnia and suicide. The second model further explored this relationship by incorporating stay-at-home measures, allowing us to differentiate between the direct effect of COVID-19 deaths and the portion of the effect that might be influenced by stay-at-home measures. The third model focused on the connection between COVID-19 deaths and the stay-at-home measures, helping to determine their role as a mediator in the relationship. To account for differences across countries and time periods, the analysis included adjustments for variations specific to each country and time frame. SEs were estimated using the delta method. The analyses were conducted using R software, version 3.6.3 (R Foundation for Statistical Computing). The methodological details, including the specific modeling strategies and estimation procedures, are provided in the Multimedia Appendix 1.

### Ethical Considerations

This study analyzed publicly available, country-wide statistics from March 2020 to February 2021 across 45 countries. The data sources included (1) Google search volumes for “insomnia” and “suicide,” (2) anonymized and aggregated cell phone location data, and (3) the COVID-19 pandemic confirmed cases and death statistics. All data were publicly accessible, nonidentifiable, and noninterventional, ensuring that no individual could be identified. Per the Medical Research Ethics Committee (Chapter VI), institutional review board approval was waived in accordance with institutional policies as the study exclusively used publicly available and anonymized data without any direct interaction or intervention [25].

## Results

Table 1 presents a comprehensive view of the notable surge in stay-at-home behaviors across various countries worldwide in response to the COVID-19 pandemic during its first, second, and third years. Among these countries, which include Australia, Brazil, Canada, Chile, India, Indonesia, Japan, Mexico, the Netherlands, Peru, Singapore, South Africa, United Kingdom, United States, and Venezuela, the adherence to stay-at-home measures remained consistent over this period. Moreover, those nations that witnessed a significant increase in stay-at-home behaviors during the first year continued to do so in either the second or third year. In contrast, there were countries where the elevation of stay-at-home behaviors did not become significant until the third year, despite the absence of a substantial increase in the initial 2 years. Noteworthy among these nations were Egypt, Qatar, New Zealand, and Taiwan.

**Figure 2:**
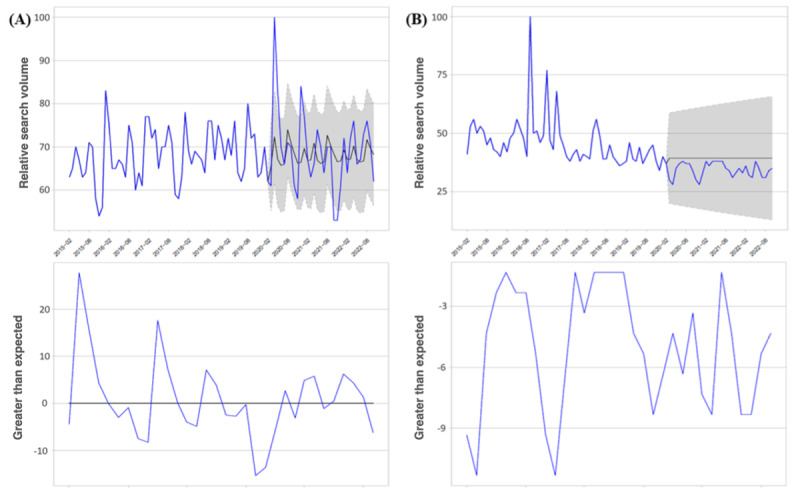
Internet search trends in Italy for (A) “insomnia” (“insomnia” in Italian) and (B) “suicide” (“suicidio” in Italian) from March 1, 2015, to October 15, 2022. Upper panels: Observed search volume for “insomnia” and “suicide” (in blue). The gray area represents the 95% CI of the expected search volume (in black) from March 1, 2020, to October 15, 2022. Lower panels: Excess observed search volume compared with the expected search volume. By using search volumes for both keywords (“insomnia” and “suicide”) from March 1, 2015, to February 28, 2020, we created a counterfactual scenario to estimate expected search volumes without the COVID-19 outbreak. Expected volumes were calculated using Hyndman and Khandakar’s algorithm for sARIMA modeling. The alteration in “insomnia” search volume was evaluated through the difference between observed and expected volumes weekly from March 1, 2020, to October 15, 2022

**Figure 3:**
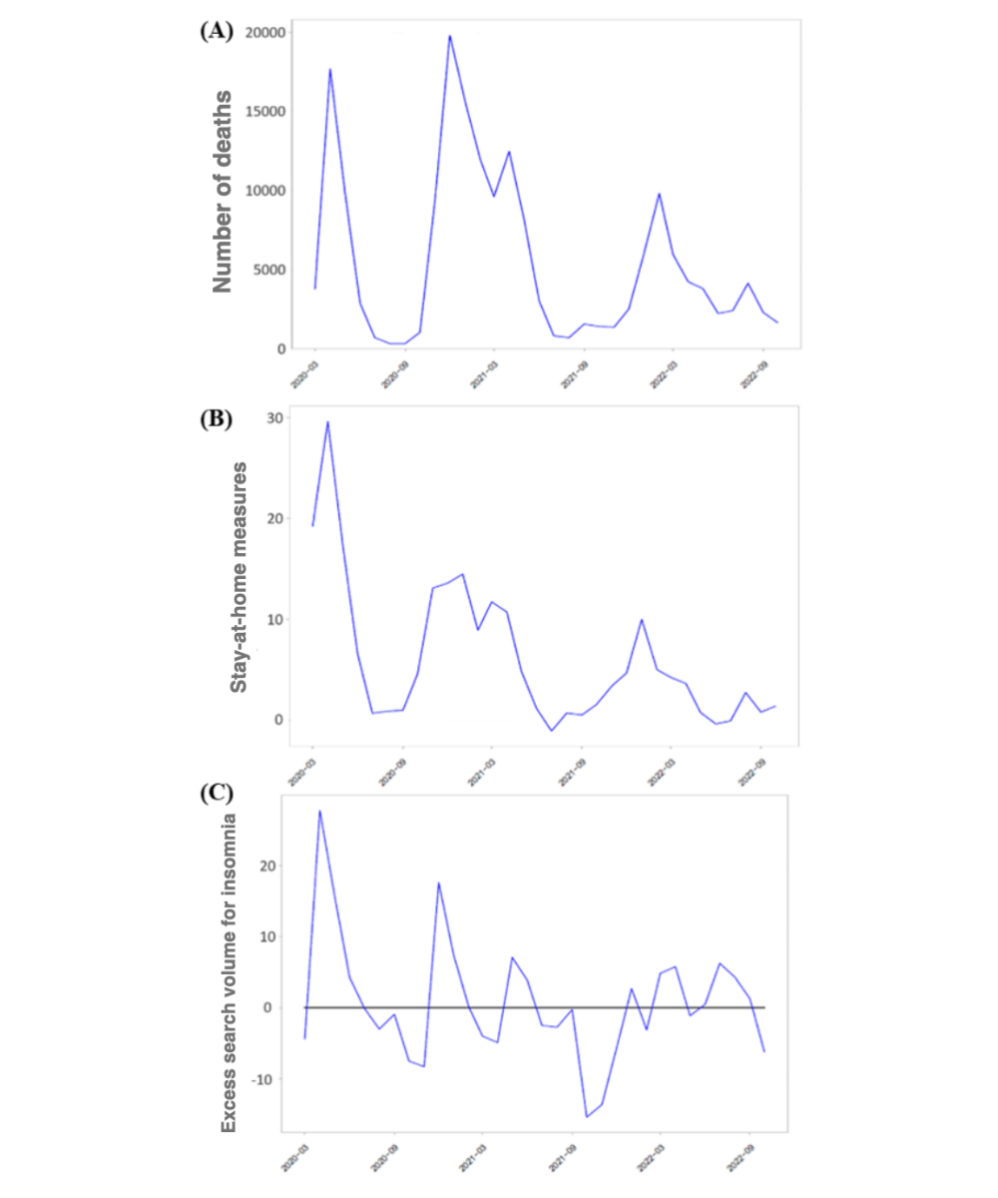
Trajectories of daily COVID-19 deaths, stay-at-home measures, and changes in internet searches for “insomnia” (“insomnia” in Italian) and “suicide” (“suicidio” in Italian) from baseline. (A) COVID-19 Death Trends: The daily count of COVID-19 deaths from March 1, 2020, to October 15, 2022. (B) Stay-at-Home Measures: The variations in stay-at-home measures from March 1, 2020, to October 15, 2022, relative to the baseline period of January 3 to February 6, 2020. (C) Mental Health Impact: Changes in internet searches for “insomnia” from baseline. The shifts in relative search volumes for “insomnia” during March 1, 2020, to October 15, 2022, compared with the baseline period of March 1, 2015, to February 29, 2020 (details are provided in Figure 2).

Although several countries experienced occasional months during the pandemic when internet searches for “insomnia” and “suicide” exceeded baseline levels, the aggregation of data over the first, second, and third years revealed no country maintaining consistently elevated levels of internet search volumes for these terms throughout the entire year. As depicted in Figure 2, Italy serves as an illustrative example for comparing observed search volumes to expected search volumes. However, Figure 3, as highlighted, reveals that certain months witnessed an increase in daily COVID-19 deaths, often accompanied by simultaneous rises in stay-at-home measures and corresponding shifts in internet searches for “insomnia” and “suicide.” This complex interplay is further depicted in Figure 3, outlining the intricate relationship among stay-at-home measures, daily COVID-19 deaths, and deviations in internet searches for “insomnia” and “suicide” from the baseline period.

The results of the causal mediation analysis are synthesized in Tables 2 and 3. The impact of COVID-19 death counts on internet search volumes for the keyword “insomnia” exhibited nuanced variations across income groups and timeframes. During the initial pandemic year (March 2020 to February 2021), significant associations emerged between COVID-19 death counts and search volumes for “insomnia” within high-income countries. In essence, a higher monthly count of COVID-19 deaths within these countries corresponded to a more pronounced surge in internet searches for “insomnia” during the same month. Moreover, the heightened search volume for “insomnia” was mediated by stay-at-home behavior, signifying substantial mediation effects. Approximately 31.9% of the overall effect was attributed to mediation by stay-at-home behaviors. However, as the pandemic progressed into the second and third years, the total effects of COVID-19 death counts on the “insomnia” search volume lost their significance within high-income countries. Conversely, throughout the 3-year study period, there was no notable impact of COVID-19 death counts on the search volume for the “insomnia” keyword in middle-income countries.

Regarding internet search volumes for the keyword “suicide,” the relationship with COVID-19 death counts revealed distinct patterns across various income groups and timeframes. In middle-income countries, both the first and second years of the pandemic exhibited noticeable negative total effects between COVID-19 death counts and the search volume for the “suicide” keyword. This indicated that, in these middle-income countries, a higher monthly count of COVID-19 deaths coincided with a reduction in internet searches for “suicide” during the corresponding month. Moreover, the observed reduction in “suicide” search volume during these years was predominantly influenced by factors other than stay-at-home behaviors, as the mediation effects were not statistically significant. Conversely, in the initial and subsequent years of the pandemic within high-income countries, there was no substantial impact of COVID-19 death counts on the search volume for the “suicide” keyword.

A noteworthy observation emerged in high-income countries during the second year, revealing a positive association with borderline statistical significance (*P*=.05) between COVID-19 death counts and the search volume for the “suicide” keyword. As the pandemic extended into its third year, a significant influence of COVID-19 death counts on the search volume for the “suicide” keyword became evident within high-income countries. Consistent with the trend observed in middle-income countries, this influence was primarily driven by alternative factors unrelated to stay-at-home behaviors, and the mediation effects did not attain statistical significance.

**Table 2:** Mediation analysis between COVID-19 death and “insomnia” search volumes, mediated by stay-at-home behaviors.

		First year (2020/3-2021/2)			Second year (2021/3-2022/2)				Third year (2022/3-2022/10)		
		Estimate	95% CI	*P* value	Estimate	95% CI	*P* value	Estimate		95% CI	*P* value
**High income**											
	Total effect	2.1×10^–4^	4.3×10^–5^ to 3.9×10^–4^	.01	1.2×10^–4^	1.0×10^–4^ to 3.4×10^–4^	.25	1.2×10^–4^		–3.2×10^–4^ to 5.4×10^–4^	.54
	Mediation effect	6.7×10^–5^	2.3×10^–5^ to 1.2×10^–4^	.004	3.4×10^–5^	–8.1×10^–6^ to 9.5×10^–5^	.13	–17		–4.9×10^–5^ to 4.5×10^–5^	.95
	Alternative effect	1.5×10^–4^	–2.2×10^–5^ to 3.2×10^–4^	.09	9.1×10^–5^	1.3×10^–4^ to 3.0×10^–4^	.38	1.2×10^–4^		–3.2×10^–4^ to 5.5×10^–4^	.54
	Proportion mediated (%)	31.9	9.8 to 127.5	.02	28.3	–194.2 to 265.7	.34	9.1×10^–1^		–90.3 to 72.3	.99
**Middle income**											
	Total effect	2.2×10^–4^	4.8×10^–5^ to 5.1×10^–4^	.10	1.0×10^–4^	–4.6×10^–5^ to 2.4×10^–4^	.16	1.2×10^–3^		–1.2×10^–4^ to 2.4×10^–3^	.06
	Mediation effect	2.1×10^–4^	9.8×10^–5^ to 3.4×10^–4^	<.001	–20	–5.2×10^–5^ to 1.5×10^–5^	.32	1.2×10^–5^		–6.6×10^–5^ to 1.1×10^–4^	.79
	Alternative effect	1.2×10^–5^	–2.4×10^–4^ to 2.6×10^–4^	.94	1.2×10^–4^	–3.1×10^–5^ to 2.6×10^–4^	.13	1.2×10^–3^		–1.2×10^–4^ to 2.4×10^–3^	.06
	Proportion mediated (%)	95.5	–360.3 to 630	.10	–15	–157.7 to 98.1	.43	1		–15.2 to 15.1	.81

**Table 3:** Mediation analysis between COVID-19 death and “suicide” search volumes, mediated by stay-at-home behaviors.

		First year (2020/3-2021/2)				Second year (2021/3-2022/2)				Third year (2022/3-2022/10)		
		Estimate	95% CI	*P* value	Estimate		95% CI	*P* value	Estimate		95% CI	*P* value
**High income**												
	Total effect	1.2×10^–5^	–1.3×10^–4^ to 1.5×10^–4^	.86	2.2×10^–4^		–9.5×10^–7^ to 4.2×10^–4^	.05	5.0×10^–4^		5.0×10^–5^ to 1.0×10^–3^	.03
	Mediation effect	–2.0×10^–5^	–5.5×10^–5^ to 7.9×10^–6^	.20	2.5×10^–5^		–1.5×10^–5^ to 8.0×10^–5^	.23	2.9×10^–7^		–3.1×10^–5^ to 3.2×10^–5^	.96
	Alternative effect	3.2×10^–5^	–1.2×10^–4^ to 1.8×10^–4^	.63	1.9×10^–4^		–1.4×10^–5^ to 4.0×10^–4^	.07	5.0×10^–4^		4.8×10^–5^ to 1.0×10^–3^	.02
	Proportion Mediated (%)	–166.6	–595.2 to 448.9	.91	11.4		–21.9 to 70.1	.26	5.8×10^–2^		–8.9 to 9.8	.95
**Middle income**												
	Total effect	–3.5×10^–4^	–6.1×10^–4^ to –9.8×10^–5^	.006	–2.6×10^–4^		–4.5×10^–4^ to –6.8×10^–5^	.01	–9.5×10^–4^		–2.3×10^–3^ to 2.6×10^–4^	.15
	Mediation effect	–5.1×10^–5^	–1.3×10–^4^ to 2.8×10^–6^	.07	3.0×10^–5^		–1.4×10^–5^ to –8.7×10^–5^	.18	–7.5×10^–6^		–9.0×10^–5^ to 6.2×10^–5^	.83
	Alternative effect	–3.0×10^–4^	–5.5×10^–4^ to –6.3×10^–5^	.02	–2.9×10^–4^		–4.9×10^–4^ to –9.5×10^5^	.008	–9.5×10^–4^		–2.3×10^–3^ to 3.0×10^–4^	.15
	Proportion mediated (%)	14.6	–1.0 to 48.9	.07	–11.5		–51.4 to 7.2	.19	0.7		–19.0 to 26.7	.86

In the sensitivity analysis, where the COVID-19 incidence rate replaced COVID-19 deaths as an alternative measure of the pandemic’s impact, the findings largely aligned with the primary results. Regarding internet search volumes for “insomnia,” the previously observed significant total effect of COVID-19 deaths in high-income countries during the first year remained statistically nonsignificant when using COVID-19 incidence rate instead. However, the proportion mediated by stay-at-home behaviors was 36.6%, closely resembling the original estimate of 31.9% (Table 2 and Table S1 in Multimedia Appendix 1). For internet search volumes for “suicide,” the sensitivity analysis yielded several statistically consistent findings. In high-income countries, the significant positive association between COVID-19 deaths and suicide-related searches in the third year remained significant when using COVID-19 incidence rate instead. Similarly, in middle-income countries, the significant negative association observed in the first year persisted across both measures. In both cases, the mediation effect via stay-at-home behaviors was not statistically significant, indicating that the observed associations were primarily driven by alternative factors (Table 3 and Table S2 in Multimedia Appendix 1). However, in middle-income countries, while COVID-19 deaths exhibited a significant negative association with suicide-related searches in the second year, this effect was no longer statistically significant when using the COVID-19 incidence rate, despite a similar negative trend. These findings highlight the robustness of the overall patterns while underscoring the nuanced differences arising from different measures of pandemic severity.

## Discussion

### Principal Findings

This study provides new insights into the evolving impact of COVID-19 on mental health across different income-level countries. In high-income countries, increased COVID-19 deaths during the first year of the pandemic were associated with a rise in “insomnia” searches, with a substantial portion of this effect mediated by stay-at-home behaviors. However, this association weakened in subsequent years. In contrast, middle-income countries exhibited a distinct pattern, where higher COVID-19 deaths were linked to a reduction in “suicide” searches during the first 2 years, independent of stay-at-home behaviors. By the third year, a significant association between COVID-19 deaths and “suicide” searches emerged in high-income countries, highlighting shifting mental health responses as the pandemic evolved.

Our study added value by investigating the correlation between the COVID-19 pandemic and mental health. Validating the outcomes of previous comprehensive meta-analyses [5] and large-scale survey [6], our work reinforced the significance of monitoring “insomnia” searches as potential indicators for emerging mental health challenges. Furthermore, this study highlighted the mediating role of stay-at-home behaviors, particularly in high-income countries, where mobility restrictions significantly contributed to increased “insomnia” searches in the first year. These findings were in line with insights derived from meta-regression analyses, emphasizing the critical role of key pandemic indicators, such as COVID-19 infection rates and reductions in human mobility, in influencing the prevalence of major depressive disorder and anxiety disorders [5]. As the pandemic evolved, however, factors such as vaccine rollout [26] and the emergence of the less fatal Omicron variant [27], contributed to a decline in both “insomnia” searches and the impact of mobility restrictions on mental health-related search behaviors. The findings suggest that public health measures and psychological responses to the pandemic were not static but changed over time, underscoring the need for adaptable mental health interventions across different pandemic phases.

### Mechanisms Driving Stay-at-Home Effects

The observed mediation effect of stay-at-home measures on “insomnia” search volumes can be attributed to multiple psychological mechanisms. Increased isolation [28], disruptions to daily routines [29], and reduced access to coping resources likely exacerbated stress levels [30], contributing to sleep disturbances. For example, isolation from social support networks may heighten loneliness and anxiety, while the lack of structured routines during prolonged lockdowns can disrupt circadian rhythms, amplifying insomnia [2,12]. Research supports the idea that increased social isolation during self-quarantining and social distancing is associated with elevated anxiety and depression levels. A longitudinal study conducted in the United States. demonstrated that individuals engaging in greater self-quarantining and social distancing experienced higher anxiety and depression during those periods, though no prospective effects were found, indicating a complex, immediate relationship between isolation and mental health [28]. Furthermore, disruptions to daily routines have been shown to significantly impact mental health outcomes. A meta-analysis of over 900,000 respondents across 53 studies revealed that disruptions in physical activity, sleep, and eating routines were positively associated with depressive symptoms, anxiety, and general psychological distress. These effects were more pronounced in regions experiencing greater pandemic severity and less governmental economic support, highlighting the role of daily routine regularity in mitigating mental health challenges during large-scale disasters [29]. Additionally, coping strategies during lockdowns played a pivotal role in shaping mental health outcomes. Evidence suggests that avoidant-emotional coping styles were linked to higher odds of anxiety, depression, and sleep problems. Conversely, problem-focused coping strategies were associated with lower odds of depression, offering a source of resilience amid the pandemic’s challenges. These findings emphasize the critical importance of fostering effective coping mechanisms to mitigate psychological distress [30]. Limited access to mental health services during lockdowns further compounded psychological distress, increasing reliance on digital platforms for coping-related information [31]. Recent studies corroborate these findings, demonstrating that such disruptions significantly influenced mental health outcomes during the pandemic [14,28-30,32,33]. These insights highlight the multifaceted nature of the psychological mechanisms at play and underscore the need to address isolation, routine disruptions, and coping resources when designing public health responses to mitigate the adverse mental health effects of future large-scale disasters.

### Income-Based Disparities

The dynamic between “suicide” search volumes and COVID-19 death counts presented intriguing variations across income groups and phases. Middle-income countries, during the first 2 years, witnessed a decrease in “suicide” searches despite higher COVID-19 death counts, aligning with the previous findings [6,31]. This pattern suggests that shared experiences and community support may temporarily buffer against suicidal ideation, particularly in times of collective crisis. Enhanced mental health interventions, financial aid, and subsidies in middle-income countries may have further mitigated immediate psychological impacts. This pattern suggests that shared experiences and community support may temporarily buffer against suicidal ideation, particularly in times of collective crisis [34]. Enhanced mental health interventions, financial aid, and subsidies in middle-income countries may have further mitigated immediate psychological impacts [32]. In contrast, high-income countries initially exhibited minimal impact on “suicide” search volumes, but a potential link surfaced during the pandemic’s second year, gaining statistical significance in the third year. This divergence may stem from structural differences, such as public health messaging strategies, which often differ in their emphasis on mental health [33]. High-income countries may prioritize broader health communication but sometimes neglect specific, localized mental health campaigns, while middle-income countries might leverage community-based outreach to foster solidarity and awareness [35].

These variations in mental health-related keyword search volumes across income groups can also be better understood through comparisons of mobility trends observed during the early stages of the COVID-19 pandemic in different socioeconomic contexts. For instance, a comparison of studies conducted in the United States (January 22, 2020, to May 11, 2020) [36] and Nigeria (February 27, 2020, to July 21, 2020) [21] reveals notable similarities and differences. Both studies used publicly available cell phone location data provided by Google and reported a significant increase in residential activity during stay-at-home orders or lockdowns. This highlights the effectiveness of government measures in reducing mobility to curb virus transmission. However, Nigeria exhibited a more pronounced decrease in non-residential activity and a sharper rebound following the easing of lockdown measures, with retail activity recovering by 14.6 percentage points after restrictions were partially lifted [21]. In contrast, the United States showed smaller increases in residential activity, reflecting more moderate changes in mobility patterns [36]. These differences might be attributed to the contrasting economic conditions of the 2 countries. The United States, as a high-income country, benefits from robust economic infrastructure, widespread digital technology access, and higher rates of remote work adoption, contributing to less drastic shifts in mobility. Conversely, Nigeria, a lower-middle–income country, has a larger informal economy and limited digital infrastructure, making prolonged lockdowns and remote work less feasible. These economic disparities likely shaped the degree and pace of mobility reductions and subsequent rebounds in each country.

However, interpreting these findings requires caution due to potential limitations in the data. The reliance on cell phone location data from Google Location History introduces the possibility of selection bias, as it only includes users who opted in to share their data. Opt-in users may differ significantly from non–opt-in users, such as being more likely to live in urban areas, possess higher levels of education, and have greater access to technology, potentially resulting in an overestimation of compliance with stay-at-home orders. In addition, in less affluent countries like Nigeria, where only about one-third of adults own smartphones, the data may not fully capture mobility trends in rural or underserved regions. Noncoverage bias could arise, as smartphone users are less likely to reside in rural areas where landlines or limited connectivity may play a larger role in daily activities. These limitations emphasize the need for careful interpretation of mobility data and the exploration of alternative methods to better capture diverse mobility trends across varying socioeconomic contexts.

### Influence of Confounding Factors

Beyond the direct effects of pandemic-related stressors, media coverage and public health campaigns likely influenced search behaviors, shaping public awareness and perceptions of mental health issues [37,38]. Increased media attention to insomnia and suicide during the pandemic may have contributed to search volume fluctuations, while targeted mental health campaigns could have directed individuals toward online resources, amplifying observed trends [38,39]. However, the influence of media is complex, positive portrayals can reduce stigma and promote help-seeking, whereas negative or fear-based narratives may discourage individuals from seeking support [37]. Social media platforms further complicate this landscape, as they serve as both sources of information and channels for misinformation, potentially shaping search behaviors in unintended ways.

Moreover, engagement with social media health campaigns follows non-linear patterns, where metrics such as likes, shares, and comments amplify message reach but do not necessarily translate into meaningful behavioral change [38]. While campaigns featuring inspirational content and peer support have been shown to foster engagement, stigmatizing narratives also garner attention, underscoring the need for carefully designed messaging that balances awareness and stigma reduction [39].

In addition to these factors, the choice of pandemic severity indicators (eg, COVID-19 deaths vs incidence rates) may introduce further variability in observed patterns. Our sensitivity analysis demonstrated that key findings remained consistent across different measures of pandemic impact, reinforcing the robustness of the overall trends while highlighting nuances in the role of alternative explanatory factors. Future studies should incorporate media dynamics and public health messaging into predictive models to disentangle their relative contributions to mental health-related search behaviors. By doing so, researchers and policy makers can develop more precise interventions that address the interplay of media influence, pandemic stressors, and public engagement with mental health resources.

### Clinical Implications

Monitoring digital search trends provides a unique opportunity to inform early intervention strategies during public health emergencies. The sharp increase in “insomnia” searches during the pandemic’s initial stages highlights the potential of digital platforms for identifying emerging mental health crises in real time. These findings can guide policy makers in implementing timely interventions, such as telehealth services or digital mental health tools, to address rising psychological distress. Moreover, differences in search behaviors across income groups suggest that interventions should be context-specific. In middle-income countries, bolstering mental health awareness and access to resources may sustain the initial gains achieved through early interventions. In high-income countries, addressing the long-term economic and psychological repercussions of the pandemic, particularly as financial support schemes phase out, will be critical. Our study underscores the importance of integrating digital epidemiology into mental health policy and practice. By leveraging real-time data from platforms like Google Trends, public health officials can enhance surveillance systems, allocate resources more effectively, and develop culturally sensitive strategies to mitigate the mental health impacts of future crises.

### Limitations

Several methodological limitations warrant consideration while interpreting our findings. First, our analysis is grounded in population-level data, which curtails our ability to explore nuances within specific subgroups, such as distinct age ranges, genders, or vulnerable populations [14]. Consequently, investigating the effects on these specific demographic groups is beyond this study’s scope. Second, while our study acknowledges the mediating role of stay-at-home behaviors in influencing search volumes, it is vital to acknowledge the presence of other indirect pathways. It is worth noting that the observed effects related to the “suicide” keyword in our study predominantly manifest as indirect effects. Third, the disparities observed between high-income and middle-income countries merit attention. For instance, in high-income countries, the initial pandemic year witnessed a significant increase in national death counts correspondingly linked to heightened internet searches for the “insomnia” keyword, whereas no such significant effect was observed in middle-income countries. This variance could be attributed to higher rates of internet penetration in high-income countries. Similarly, countries with more extensive internet access, particularly those with widespread smartphone usage, are likely to yield more representative stay-at-home data extracted from the Google Location History database. Lastly, while the first and second years of the COVID-19 pandemic each spanned complete 12-month periods (from March 1, 2020, to February 28, 2021, and from March 1, 2021, to February 28, 2022, respectively), the third year was limited due to the cessation of open access data release from the Google Location History database. This constrained the scope of our investigation for the third year, encompassing only the timeframe from March 1, 2022, to October 15, 2022.

### Conclusions

In conclusion, our study unveiled the intricate interplay between the COVID-19 pandemic, mental health, and internet search behaviors, highlighting the evolving dynamics across income levels and time. These insights underscored the imperative for tailored interventions and ongoing support to address the multifaceted mental health challenges stemming from the pandemic.

## Appendix

Multimedia Appendix 1 Additional material.

## Abbreviations

PM: proportion mediated
sARIMA: seasonal autoregressive integrated moving average

## Data Availability

The datasets analyzed during this study are available online, including the Google Trends, the Google COVID-19 Community Mobility Reports [18], and the COVID-19 Dashboard [22].
