# Supplementary material for: Search Volume of Insomnia and Suicide as Digital Footprints of Global Mental Health During the COVID-19 Pandemic: 3-Year Infodemiology Study
Source: J Med Internet Res. 2025 Apr 17;27:e67646. doi: 10.2196/67646 (PMC12046254; doi:10.2196/67646)
Supplement: Multimedia Appendix 1 [file jmir_v27i1e67646_app1.docx]

**Multimedia Appendix #1**

**Technical details for building the mediation models**

Causal mediation analysis involved constructing three regression models with the following dependent variables:

Model 1: $E\left[ \left( Google search volumes for "\text{insomnia"} \right)_{ct} \right| COVID-19 \mathrm{deaths}_{ct}]= \beta_{01}+ \beta_{1} \times COVID-19 \mathrm{deaths}_{ct}$

Model 2: $E\left[ \left( Google search volumes for \text{insomnia} \right)_{ct} \right| COVID-19 \mathrm{deaths}_{ct}, stay-at-home \mathrm{measures}_{ct}]= \beta_{02}+ \beta_{2}\times COVID-19 \mathrm{deaths}_{ct}+ \beta_{3}\times stay-at-home \mathrm{measures}_{ct}$

Model 3: $E\left[ stay-at-home \mathrm{measures}_{ct} \right| COVID-19 \mathrm{deaths}_{ct}]= \beta_{4}\times\mathrm{COVID}-19 \mathrm{deaths}_{ct}+\gamma_{c}+\delta_{t}$

Models 1 and 2 employed fixed-effects panel regression [21] with search volumes for "insomnia" and "suicide" as dependent variables, respectively. The inclusion of fixed time or group effects was determined by the standardization of search volume by expected search volume for each country. Model 3 included fixed time and group effects. Coefficient estimates for mediation analysis are provided in Tables 2 and Table 3. Subsequent causal mediation analysis utilized these models. The coefficient of COVID-19 deaths (β1) in Model 1 measured the total effect of COVID-19 deaths on the search volume for "insomnia" (a similar approach applied to "suicide"). The coefficient of COVID-19 deaths (β2) in Model 2 represented the alternative effect, denoting the impact of COVID-19 deaths on the search volume for "insomnia" not mediated by stay-at-home measures. The product of the coefficient of stay-at-home measures in Model 2 (β3) and the coefficient of COVID-19 deaths in Model 3 (β4) quantified the mediation effect. The former coefficient indicated the influence of stay-at-home measures on search volumes, while the latter coefficient demonstrated the connection between COVID-19 spread and stay-at-home measures. This product elucidated the mechanism by which COVID-19 spread impacted the population's mental health via stay-at-home measures (mediation effect). Standard errors were estimated using the delta method. The analyses were conducted using R software, version 3.6.3 (R Foundation for Statistical Computing).

**Table S1. Mediation analysis between COVID-19 incidence rate and "insomnia" search volumes, mediated by stay-at-home behaviors.**

|  | First year (2020/3-2021/2) | | | Second year (2021/3-2022/2) | | | Third year (2022/3-2022/10) | | |
| --- | --- | --- | --- | --- | --- | --- | --- | --- | --- |
|  | Estimate | 95%CI | P value | Estimate | 95%CI | P value | Estimate | 95%CI | P value |
| **High income** |  |  |  |  |  |  |  |  |  |
| Total effect | 40.9 | (-69.8, 155.4) | 0.480 | 15.1 | (-2.5, 32.6) | 0.094 | 8.8 | (-10.9, 28.6) | 0.402 |
| Mediation effect | 29.2 | (8.4, 58.8) | 0.001 | 0.2 | (-1.4, 2.0) | 0.784 | 0.7 | (-1.2, 3.4) | 0.510 |
| Alternative effect | 11.7 | (-99.8, 127.5) | 0.852 | 14.9 | (-3.4, 32.5) | 0.102 | 8.1 | (-12.0, 28.2) | 0.442 |
| Proportion Mediated (%) | 36.6 | (-708.3, 556.7) | 0.480 | 0.6 | (-28.2, 23.2) | 0.814 | 3.3 | (-91.8, 113.1) | 0.664 |
| **Middle income** |  |  |  |  |  |  |  |  |  |
| Total effect | -100.3 | (-212.9, 10.1) | 0.068 | -36.6 | (-86.2, 14.3) | 0.140 | 25.8 | (-121.3, 166.5) | 0.736 |
| Mediation effect | 45.6 | (-2.1, 94.6) | 0.062 | -0.3 | (-15.2, 14.1) | 0.958 | -8.6 | (-27.5, 9.9) | 0.336 |
| Alternative effect | -145.8 | (-252.1, -47.6) | 0.012 | -36.3 | (-89.0, 18.6) | 0.160 | 34.4 | (-114.2, 176.9) | 0.642 |
| Proportion Mediated (%) | -39.7 | (-661.3, 138.2) | 0.130 | 0.3 | (-132.7, 87.8) | 0.982 | -4.3 | (-197.8, 249.9) | 0.820 |

**Table S2. Mediation analysis between COVID-19 incidence rate and "suicide" search volumes, mediated by stay-at-home behaviors.**

|  | First year (2020/3-2021/2) | | | Second year (2021/3-2022/2) | | | Third year (2022/3-2022/10) | | |
| --- | --- | --- | --- | --- | --- | --- | --- | --- | --- |
|  | Estimate | 95%CI | P value | Estimate | 95%CI | P value | Estimate | 95%CI | P value |
| **High income** |  |  |  |  |  |  |  |  |  |
| Total effect | 37.9 | (-56.2, 126.9) | 0.400 | 13.8 | (-3.2, 31.4) | 0.118 | 33.7 | (9.7, 57.7) | 0.006 |
| Mediation effect | -8.7 | (-25.1, 3.6) | 0.186 | 0.1 | (-1.1, 1.6) | 0.806 | -0.1 | (-2.0, 1.7) | 0.910 |
| Alternative effect | 46.6 | (-46.9, 135.2) | 0.302 | 13.7 | (-3.4, 31.5) | 0.126 | 33.9 | (9.8, 57.7) | 0.006 |
| Proportion Mediated (%) | -10.1 | (-238.9, 264.2) | 0.512 | 0.4 | (-18.6, 30.9) | 0.828 | -0.1 | (-8.3, 6.1) | 0.912 |
| **Middle income** |  |  |  |  |  |  |  |  |  |
| Total effect | -120.1 | (-217.9, -27.1) | 0.008 | -64.3 | (-136.5, 8.1) | 0.102 | 20.8 | (-124.6, 157.7) | 0.732 |
| Mediation effect | -9.5 | (-28.6, 2.4) | 0.174 | 11.2 | (-7.6, 34.6) | 0.236 | 5.9 | (-12.0, 24.1) | 0.536 |
| Alternative effect | -110.5 | (-211.5, -14.6) | 0.018 | -75.5 | (-152.9, 6.7) | 0.072 | 14.8 | (-131.6, 146.7) | 0.830 |
| Proportion Mediated (%) | 6.8 | (-2.6, 39.4) | 0.178 | -14.8 | (-144.2, 80.6) | 0.294 | 2.9 | (-148.6, 265.9) | 0.880 |
